# Factors influencing general practitioners decisions to refer Paediatric patients to the emergency department: a systematic review and narrative synthesis

**DOI:** 10.1186/s12875-020-01277-9

**Published:** 2020-10-16

**Authors:** Ciara Conlon, Emma Nicholson, Beatriz Rodríguez-Martin, Roisin O’Donovan, Aoife De Brún, Thérѐse McDonnell, Gerard Bury, Eilish McAuliffe

**Affiliations:** 1 Centre for Interdisciplinary Research, Education and Innovation in Health Systems (IRIS), UCD School of Nursing, Midwifery & Health Systems, University College Dublin, Belfield, Dublin 4 Ireland; 2 Faculty of Health Sciences, University of Castilla-La Mancha, Avd. Real Fabrica de Sedas s/n. 45600 Talavera de la Reina, Toledo, Spain; 3 School of Medicine, Health Sciences Centre, University College Dublin, Belfield, Dublin 4 Ireland

**Keywords:** Decision-making, Referrals, Paediatric, Unscheduled healthcare, Non-clinical factors

## Abstract

**Background:**

Clinical guidelines are integral to a general practitioner’s decision to refer a paediatric patient to emergency care. The influence of non-clinical factors must also be considered. This review explores the non-clinical factors that may influence general practitioners (GPs) when deciding whether or not to refer a paediatric patient to the Emergency Department (ED).

**Methods:**

A systematic review of peer-reviewed literature published from August 1980 to July 2019 was conducted to explore the non-clinical factors that influence GPs’ decision-making in referring paediatric patients to the emergency department. The results were synthesised using a narrative approach.

**Results:**

Seven studies met the inclusion criteria. Non-clinical factors relating to patients, GPs and health systems influence GPs decision to refer children to the ED. GPs reported parents/ caregivers influence, including their perception of severity of child’s illness, parent’s request for onward referral and GPs’ appraisal of parents’ ability to cope. Socio-economic status, GPs’ aversion to risk and system level factors such as access to diagnostics and specialist services also influenced referral decisions.

**Conclusions:**

A myriad of non-clinical factors influence GP referrals of children to the ED. Further research on the impact of non-clinical factors on clinical decision-making can help to elucidate patterns and trends of paediatric healthcare and identify areas for intervention to utilise resources efficiently and improve healthcare delivery.

## Background

General practitioners (GPs) are often the first point of contact to healthcare, acting as a gateway to emergency, specialist, or other secondary care services in many countries [1, 2]. Rising utilization of emergency departments (ED) has been recorded internationally, leading to increased attention to reducing demand on emergency services [3, 4]. This debate is commonly framed in terms of the appropriateness of visits, and children have been identified as high users of the ED with conditions treatable in primary care [3, 5]. GPs contribute substantially to ED attendance rates through referrals. In the UK, GP referrals account for 21% of emergency admissions annually [6], while in Australia 8% of all presentations to the ED are referred by a GP [7]. In Ireland, referrals account for approximately 37–38% of paediatric ED attendances in 2015 [8]. However, little is known about the decision-making process behind GP referrals to the ED, particularly when it comes to children [9].

Significant variation in GP referral patterns to secondary care has been recorded, however the reasons are manifold and not fully understood [6, 10, 11]. Referrals are highly complex and present challenges to GPs, particularly in relation to children, with decisions made in a time pressured manner [6, 7]. While clinical aspects of the presenting condition are fundamental to the decision to refer, a multitude of other factors influence GP decision-making, including a complex interplay of clinical and non-clinical factors relating to the GP, the patient and health system considerations [10, 12].

Non-clinical factors have been identified in previous literature, although this is predominantly in relation to specialist and other secondary care services and not focused exclusively on paediatric patients [11, 13–15]. These include characteristics of the GP such as level of training received, or clinical experience [13]. This may be particularly pertinent with paediatric patients where lack of exposure to paediatric training may lead to a loss of confidence in treating children [7, 14]. Risk aversion, tolerance of uncertainty and the interaction between doctor and patient is also significant to understanding referral rates [15–17]. Patients’ expectations and pressure to refer may have some bearing, with parental requests reported as influencing referral decisions in a number of studies [2, 18, 19]. Finally, structural considerations such as the organisation of the health system, accessibility of specialist care, and waiting lists may also affect the GP’s referral rates [10, 20]. Insight into the trends and patterns of GP referrals is critical to informing health system policy and management, particularly in the context of rising pressure on EDs [7].

There is a paucity of research regarding the influence of non-clinical factors on GP decision-making regarding referrals. This review aims to address this by conducting a systematic review exploring the non-clinical factors that may impact a GP’s decision to refer a paediatric patient to the ED.

## Methods

A systematic review was conducted to establish the non-clinical factors that influence the decision-making of GPs when referring paediatric patients to the ED. The review was conducted following the PRISMA framework [21]. The protocol was registered with the International Prospective Register of Systematic Reviews (PROSPERO, registration no. CRD42020145233) [22].

### Search strategy

Search terms were developed following a limited search of the databases Medline (Pubmed), CINAHL, Web of Science, Embase and PsycINFO. The search terms utilized are displayed in Additional File 1 (see Additional file 1). Five databases were used: Medline (Pubmed), CINAHL, Web of Science, Embase and PsycINFO. A modified search term strategy was employed for a secondary search of Google Scholar, of which the first 10 pages were selected and reviewed for relevance, and Lenus (an Irish database). References of included articles were also screened. At the outset of the search, the time span covered articles published in English from August 2010 until July 2019. However, as the initial screening produced a small number of studies and to ensure a broader literature was included in the review, the date parameter was extended to capture literature from 1980.

**Fig. 1:**
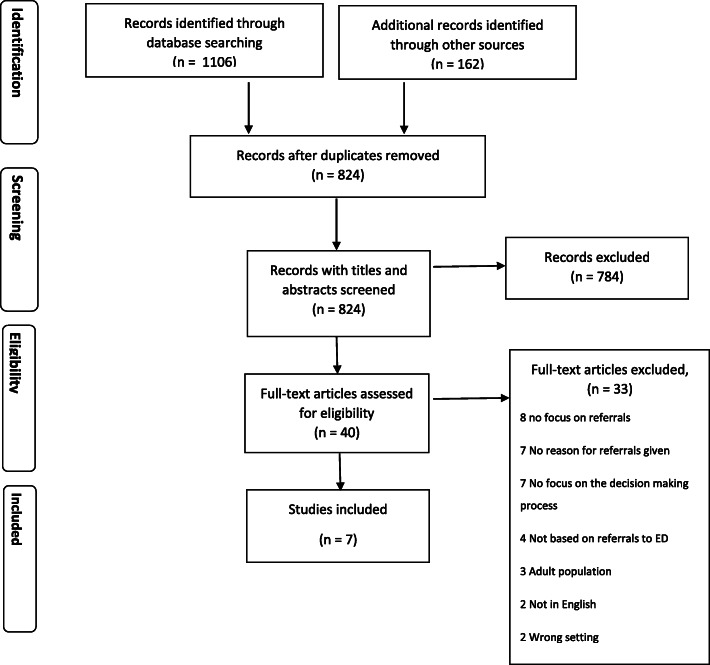
Prisma Flow Diagram

### Inclusion and exclusion criteria and screening

The search strategy and screening process is documented in the PRISMA diagram [21] in Fig. 1. Both qualitative and quantitative primary studies published in English that aimed to analyse non-clinical factors that influence GP decision-making when referring paediatric patients to emergency services were included. Studies were excluded if they focused on adult populations only and were expert opinions or editorials. Title and abstracts of articles obtained from the searches were screened independently by two researchers (EN & CC) using the online review management software Covidence™ [23]. This was then followed by independent full text review by two researchers (EN & CC) and any conflicts were discussed and resolved.

### Data extraction and quality assessment

Data was extracted by the primary researcher (CC) and a second researcher independently extracted data from three included articles (EN). Characteristics of the included studies are displayed in Tables 1 and 2. All included studies had 100% complete data sets.

**Table 1:** Details On Included Studies

Author & Date	Health System	GP Population		Paediatric Population				
		*Sample Size*	*Type of Work*	*Sample Size*	*Age*	*Gender*	*Insurance Status*	*Reason for Seeking Unscheduled Healthcare*
*Barwise- Munro, Morgan & Turner, 2018*	Universal Care	Referring GPs (7), Receiving clinicians (10)	Referring GPs: Family doctors (2), GP’s working in ED (3) & Out of Hours services (3) Receiving Clinicians: All working in the acute medical paediatric unit. Clinicians who are either in their foundation year, specialist trainees or associated specialists.	10 children and their parents	Between 5wks – 10 yrs.	9 mothers, 1 father	n/a	Level of consciousness Acutely unwell Breathing problems Breathing problems Fever and shaking Allergic reaction seizure
*Brousseau, D.* et al*, 2011*	Private or Public	20 PCPs	75% were paediatricians, 25% were family practice, general practice and internal medicine.	26 parents of children	< 2: 50%, 2–4: 23%, > 4: 27%	21 female and 5 male parents’; 14 girls	Public: 69% Private: 15% Unknown: 8% None: 8%	Fever with or without vomiting and diarrhoea: 6 (23%) Pain (oral, tympanic): 6 (23%) Breathing problems (wheezing, coughing, etc): 5 (19%) Rash: 2 (8%) Laceration: 2 (8%) Bump on the head: 2 (8%) Nosebleed: 1 (4%) Minor trauma: 1 (4%) Swollen eye: 1 (4%)
*Dale, J.* et al*, 1995*	Universal care	6 GPs; 31 ED Physicians	GPs: local General Practitioners; ED Physicians: 27 senior house officers, 3 registrars and 1 Senior registrar	< 16 yrs. n: 913 (19.7%); Overall *n* = 4641		Not reported	n/a	Not reported
*Haimi, M.* et al*, 2018*	Treatment at an ED is free on referral from a GP	15 Physicians working in telemedicine	All paediatric specialists, 7 with subspecialty. Professional experience range: 4 -30 yrs. (m: 19 yrs), paediatric telemedicine experience range: 5–9 yrs.	Not reported	Not reported	Not reported	n/a	Not reported
*Kini,N.M & Strait, R., 1998*	Private or Public	364 PCP’s	Primary Care Physicians	364	Not reported	Not reported	52% enrolled in a health maintenance organisation (HMO) which requires prior approval by PCP for non-urgent visits to the ED	Fever, minor trauma, and respiratory and gastrointestinal disorders
*Orimadegun, A.E.* et al*, 2008*	Not reported	974 referral letters written by various healthcare professionals	Letters were written by physicians (69.2%), registered nurses (21.3%), hospital assistants (2.1%), traditional birth attendants (0.4%), and non- health workers (0.3%). The identity of the writers of 65 letters (6.7%) could not be defined	974	1 day - 16 years median age of 43 months	Male: 568; Female: 406	Not reported	Not reported
*Rhodes, K.* et al*, 2012*	Private or Public	26 specialists and 14 PCP’s	Specialty physicians Subspecialty type: Allergy/immunology (2) Cardiology (1) Developmental paediatrics (4) Emergency medicine (1) Neurology (3) Ophthalmology (2) Orthopaedic surgery (3) Otolaryngology (3) Paediatric intensive care (1) Physical medicine/rehabilitation (1) Psychiatry (3) Pulmonary diseases (1) Radiology (1); Primary care physicians: Adolescent medicine (1) Family medicine (1) General paediatrics (12)	Not reported	Not reported	Not reported	Publicly insured	Not reported

**Table 2:** Study Design, Methods And Factors That Influenced Decision Making

Author & Year	Country	Aims & Rationale	Study Design, Data Collection & Analysis	Sampling Strategy	Non-clinical Factors influencing behaviour and/or decision making/ Preferences elicited
Barwise- Munro, Morgan & Turner, 2018	U. K	Explore reasons why children might be more likely to be admitted to hospital, in an emergency unscheduled manner, rather than be observed at home by their caregiver or primary care team	Qualitative, semi structured interviews, thematic analysis	Convenience Sampling	•Parental anxiety and differing perceptions of illness may influence the decision to admit by most referring clinicians. •Referring clinicians reported they often err on the side of caution: “*If I was uncertain, then that means I am not happy to send them home, by definition”* •Clinicians referred to their *“Gut Instinct”* when deciding to send a patient to be admitted •Doctors stated they were more likely to refer those of lower socio-economic status •Previous admission history would influence decision •Time of day: more likely to refer later in the day than in the morning •A small number referred to their own paediatric experience a lack of which would impact on their confidence in treating
Brousseau, D. et al., 2011	U.S.A	Understand parental decisions to seek care for their children and physician perceptions of parents’ decisions to seek non-urgent emergency-department care.	Qualitative, in-depth interviews, Grounded Theory	Purposive sample of parents triaged as non- urgent in hospital emergency department on preselected days and PCP’s of same children	•PCP’s stated parent’s perception of severity of illness justified a visit to ED •PCP’s reported that they prefer to trust the instinct of parents and accept the judgements of parents and refer to the ED rather than take a risk on the child’s health. If they were unable to see a child they would refer to the ED if the parent described the child as being in severe pain, “inconsolable” or “very irritable, regardless of whether or not medication was tried before the ED visit. *“I would rather be wrong 100 times than make a mistake”* •PCP’s referred patients to the ED for resources such as tests and treatments not available in primary care offices. Perceived need of sutures, laboratories, and nosebleed cauterization resulted in immediate referral. •PCP’s did not believe ED compromised continuity of care however some differed in what they considered appropriate urgent care centres; paediatricians tended towards paediatric urgent care centres, while family practitioners were happy to utilize any urgent care services.
Dale, J. et al., 1995	U.K.	Compared the process and outcome of ‘primary care’ consultations undertaken by senior house officers, registrars and general practitioners in an accident and emergency department	Prospective controlled intervention study, administrative data, statistical analysis	Physicians: vocationally trained GPs recruited with preference to those who had recently completed training and flexible availability. Patients: stratified random sample of those assessed at nurse triage in A&E dept. with problems that could be treated in a primary care setting.	Primary care consultations made by sessionally employed GP’s in the ED resulted in less utilization of investigative and specialist resources. This included reduced rates of investigations such as radiography, prescriptions of antibiotics and referrals to specialist and outpatient clinics.
Haimi, M. et al., 2018	Israel	Explored the experiences, attitudes, and challenges of the physicians in a Paediatric Telemedicine Service operated in Israel, and explored whether the doctors are using non-medical factors (not related to the medical problem), when making the clinical decisions in this setting and if so, to identify and describe these factors.	Qualitative, semi-structured interviews, thematic	Random sample of those who currently work at the telemedicine service, or worked there in the past five years	•Doctors reported a *“moral conflict”* between offering good service to parents, who may apply pressure for a referral to the ED in order to avoid incurring cost, and adherence to best medical guidelines •When deciding whether to refer to the ED, doctors drew on their impression of the parent’s health literacy. They reported considering the parent’s ability to understand and follow instructions and capability to adequately provide care and recognise a worsening condition. This was especially important when the patient was unfamiliar to them. •GP’s relied on intuition and “rule of thumb” protocols when diagnosing patients •Those doctors perceived to be of a lower socio-economic status - judged through place of residence, distance from medical centre and language used - were referred to the ED more often •Doctor’s also took into account the accessibility and opening hours of medical centres, and referred those living in isolated areas to the ED more frequently •Doctor’s reported referring more often on weekends (which fall FRI- SAT in Israel) especially Fridays •Doctors reported considering legal implications and a fear of lawsuits as an influence on their decision to refer or not
Kini,N.M & Strait, R., 1998	U.S.A	Evaluated the pattern and reasons for non-urgent use of the paediatric emergency department during regular office hours and why primary care physicians approved such visits	Prospective, cross -sectional observational study, administrative data, χ2 method	Patients triaged as nonurgent in a paediatric ED from June to November 1994 between 6.30 am Patients triaged as non- urgent in a paediatric ED	•PED visits were approved for different reasons before and after 3.30 pm. Before 3.30 pm, visits were most commonly approved for being medically urgent. After 3.30 pm, the most frequent reason was full office schedule. •Physicians were more likely to deny a visit to the PED earlier in the day; 24/40 denials occurred before noon while 56/151 of approvals occurred before noon. •Approvals were more likely for patients who were younger, particularly 2 yrs. old or less, and for those presenting with fever or trauma.
Orimadegun, A.E. et al., 2008	Nigeria	Evaluated the quality of the contents of referral letters received at the paediatric emergency unit of the University College Hospital, Ibadan, Nigeria.	Prospective descriptive study, descriptive statistics	All referral letters were examined using a structured questionnaire.	•A worsening condition was the most frequently stated reason (17.8%) for writing a referral letter •Lack of funds to continue treatment (17.1%), lack of facilities (14.5%) and lack of expertise (10.4%) were all cited as reasons for referrals •15.8% of referrals were due to a parents’ request (15.8%)
Rhodes, K. et al., 2012	U.S.A	Explored factors, including the role of ED referrals, associated with specialists’ willingness to accept patients covered by Medicaid and the Children’s Health Insurance Program (CHIP)	Qualitative, semi-structured interviews, Qualitative iterative coding	Purposive sampling of physicians from across specialty areas that research suggested were in high demand, short supply, or both. Identified through combination of medical provider referrals (using a snowball technique) and the state licensure	Primary Care Physicians stated they used the ED as a *“middle man”* in order to ensure publicly insured (Medicaid) patients get access to outpatient specialty care and refer them to the ED to facilitate their access to specialists

Each study was quality assessed independently by two researchers (EN & CC) using the Mixed Methods Appraisal Tool (MMAT) [24]. The MMAT provides a framework for appraising quantitative, qualitative, and mixed method studies for methodological quality and rigor, addressing sources of data, analytical process, appropriateness of measurements, selection or researcher bias. Included studies scored moderate to high quality (≥50%). No studies were excluded based on their MMAT score. Detailed scoring is displayed in Additional file 2 (see Additional file 2).

## Results

Seven published studies were included in the systematic review [25–31]. The countries of origin were: U. S (*n* = 3), U. K (*n* = 2), Nigeria (*n* = 1) and Israel (*n* = 1). Studies conducted in the U. S and U. K make up the majority of the included studies therefore a hybrid private/public and universal healthcare systems are mostly represented. One study conducted in Israel outlines that ED visits are free when referred by a GP [25]. The study carried out in Nigeria does not report details on the health system [27]. Four studies [25, 26, 29, 31] utilized a qualitative methodological approach and the remaining three were quantitative [27, 28, 30]. Population sizes varied significantly; four studies had fewer than 50 participants [25, 26, 29, 31], one had 364 [30], and the remaining two just below 1000 participants [27, 28]. Further information is displayed in Table 1.

### Factors that influence GPs decision-making when making referrals

Factors influencing GPs’ decision-making and preferences elicited are represented in Table 3.

**Table 3:** Factors That Influenced Gp Decision-Making When Referring Paediatric Patients To The Ed

PATIENT-LEVEL FACTORS	
*Parental Anxiety and perception of illness*	•Parental anxiety had an influence on decision to refer [25, 29]. •Parental perceptions of the severity of illness may influence the decision [26, 29]. •GP’s trusted parents’ instincts and judgements of severity rather than take a risk on the child’s health, especially if they were unable to see the child. Referrals were more likely if parents stated the child was inconsolable or in severe pain, even when parents had not tried medication [26].
*Parental pressure to refer*	•GPs reported parent pressure for referral in order to avoid incurring cost, leading to what doctors reported as a *“moral conflict”* between offering good service to patients and adherence to best medical guidelines [25]. •15.8% of referrals in one study were due to a parent request [27].
*Parental Capability*	•GPs considered the parent’s health literacy, in order to judge their ability to understand, follow instructions and capability to adequately provide care and recognise a worsening condition. This was especially important when the patient was unfamiliar to them [25].
*Child’s age & medical history*	•GPs were more likely to approve a referral to the ED for younger patients especially those under two years old and for presentations of fever or trauma [30]. •GPs considered the child’s previous admission history when making a decision [26].
*Socio-Economic Status*	•GPs referred those of lower socio-economic status more frequently [25, 29]. •In one study GPs reported their perception of socio-economic status was based on place of residence, language and distance from medical centre [25].
GP-LEVEL FACTORS	
*Risk aversion*	•GPs reported they often err on the side of caution by referring rather than risk a child’s health [26, 29]. •GPs stated they referred to their gut instinct, intuition and rule of thumb protocols when diagnosing patients [25, 29]. •A small number of GPs stated a lack of paediatric training would impact on their confidence [29]. •The legal implications and fear of lawsuits was reported as an influence on their decision to refer or not [25]. •On the other hand, GPs temporarily employed in the ED who carried out primary care consultations were less likely to utilize investigative and specialist resources, including radiography, prescriptions of antibiotics and referrals to specialist and outpatient clinics [28].
*Preference for Referral destination*	•In one study featuring both paediatricians and general family practitioners, paediatricians were more likely to refer to paediatric urgent care centres, while general family practitioners were happy to refer to any urgent care services [26].
SYSTEM-LEVEL FACTORS	
*Time of Day & Distance from ED*	•GPs in one study were more likely to refer later in the day than in the morning [29] while in another GPs gave approval for ED visits for different reasons before and after 3.30 pm [30]. Visits were most commonly approved for being clinically urgent before 3.30 pm and after 3.30 pm a full office schedule was the most common reason cited [30]. Denial of a visit to the ED was more likely earlier in the day; 24/40 denials occurred before noon while 56/151 of approvals occurred before noon [30]. •Doctor’s reported referring more often on weekends (Friday-Saturday in Israel) especially Fridays [25]. •Patients accessibility and opening hours of medical centres were a consideration for GPs, and those living in isolated areas were referred to the ED more frequently [25].
*Access to resources and diagnostics unavailable in primary care settings*	•Lack of funds to continue treatment (17.1%), lack of facilities (14.5%) and lack of expertise (10.4%) were all reported as reasons for referrals [27]. •Patients were referred in order to access resources such as tests and treatments unavailable in primary care offices. Immediate referral was given for perceived need of sutures, laboratories, and nosebleed cauterization [26]. •The ED was used as a *“middle man”* in order to ensure publicly insured patients get access to outpatient specialty care and GPs refer them to the ED to facilitate their access to specialists [31].

### Factors relating to patients

#### Parental/ caregivers influence

Parents and/or caregivers influence featured in four studies [25–27, 29]. GPs reported parental anxiety as an influencing factor, with higher levels of perceived anxiety prompting GPs to make the decision to refer [25, 29]. Perceived level of parents’ health literacy and capability to recognise worsening signs of their children’s condition and ability to provide care were reported [25]. GPs considered the parents’ perception of severity of illness, and in one study stated they trusted parents’ instincts about the deterioration of a child’s health and accepted their judgement that it warranted an ED visit [26]. Parental pressure to refer was reported in two studies [25, 27]. In the study conducted in Nigeria, parental request accounted for 15.8% of referrals [27]. GPs in another study stated this generated a *“moral conflict”* between pleasing and/or reassuring parents, and adherence to best medical practices [25].

#### Socio-economic status

Patients of lower socio-economic status were more likely to be referred to the ED in two studies [25, 29]. In one study this was attributed to providing financial help to parents as, in the Israeli context, attendance at the ED without a GP referral is paid out of pocket [25]. In one U. S study, patients with public health insurance were referred to the ED to access specialty care [31].

#### Age & Previous History

While not statistically significant, patients who were two years old or younger were more likely to be approved for a referral for a non-urgent ED visit and GPs considered the child’s previous admission history [30].

### Factors relating to GPs

#### Risk aversion

*“Erring on the side of caution”* was reported by GPs who felt it was preferable to refer to the ED rather than risk patient’s health [26, 29]. GPs stated they like to be completely comfortable in sending a child home [29]. GPs cited relying on their *“gut instinct*” and *“rule of thumb”* protocols when considering referral [25, 29]. They reported practicing defensive medicine by considering legal implications, such as the risk of incurring lawsuits [25]. On the other hand, GPs working in the ED setting were less likely to utilize specialist services such as radiography or microbiology investigations, prescribe medications or refer to outpatient services compared to ED staff [28].

#### Preference for referral destination

A study with both paediatricians and GPs found that paediatricians held a preference for paediatric urgent care centres, while GPs were happy to refer to any urgent care centre [26].

### System level factors

#### Time of day & Distance from ED

Findings in three studies indicated time of day was a factor when referring [25, 29, 30]. Children were more likely to be referred on the weekends in one study [25], and in another GPs approved a significantly higher proportion (58%; *P* < 0.01) of non-urgent ED visits due to “*full office schedule”* after 3.30 pm [30]. Before 3.30 pm, the most common reason was medical urgency [30]. Furthermore, GPs were more likely to deny rather than approve a non-urgent visit before noon [30]. GPs reported considering accessibility to primary services for their patients, and were more likely to refer those living in isolated areas [25].

#### Resource need

The lack of certain resources within primary services such as tests, treatments, expertise and funds was also reported [26, 27]. GPs immediately referred patients for perceived need of sutures, cauterizations and access to laboratories [26]. In the study carried out in Nigeria, a lack of funds to continue treatment (17.1%), lack of facilities (14.5%) and lack of expertise (10.4%) were reported as reasons for referrals [27]. Finally, GPs utilized the ED as a pathway to access outpatient specialty care for children who had public health insurance in the face of long waiting lists for specialists [31].

## Discussion

This systematic review makes a unique contribution due to its explicit focus on the non-clinical factors that impact GP decision-making about referrals of children to the ED. The literature synthesised suggests that along with clinical factors, non-clinical considerations relating to GPs, patients and health systems play a role in the decision-making process of GPs. While it may not be fully possible to disentangle the non-clinical issues from the clinical, it is worth isolating and examining them to understand how they influence referral decision-making. Variation of referral patterns and rates has drawn attention for some time now, although reasons for this are not fully understood, indicating they are varied, idiosyncratic and integral to the context. One of the notable findings is the small number of included studies, despite the inclusion of a broad date range, suggesting the impact of non-clinical factors on the decision-making process of GPs when referring children to the ED is an under-researched area.

As highlighted by this review, a myriad of complex factors beyond purely clinical considerations impact the GPs decision to refer. While some factors are applicable to both adult and children populations, certain factors may be more pertinent in the case of children. Parental influence featured prominently, reflecting previous research on parental or patient pressure to refer [2, 12, 15, 18, 19, 32]. When seeking unscheduled healthcare, parental anxiety may be considerably heightened leading them to seek reassurance [18, 25, 29]. This can stem from an obligation of responsibility for their children’s wellbeing and unwillingness to take risks, particularly among those who have had previous traumatic health experiences, dealing with symptoms they are unfamiliar with, or with younger children who cannot verbalize the source of discomfort or pain [33] .

Attending a primary care service before presenting to the ED is not compulsory, however in certain contexts, such as Ireland, a GP referral removes the cost of attending an ED. Parents may feel it is expedient to go beyond primary care services to emergency departments, due to their perception of urgency and an assumption that ED offers higher quality of care [34–36]. This review offers some insight regarding GPs reaction to parental request; while in one study GPs reported trust in parents’ instincts regarding their children’s health status, in another it generated a *“moral conflict”* for GPs [25, 26]. Consideration of a parent’s request for a referral shows respect for parent’s wishes regarding their children’s care [2]. Participation in medical decision- making improves quality of care and health outcomes [37], and has been shown to improve parents’ satisfaction and linked to reducing unnecessary antibiotic use for children [38]. On the other hand, GPs must balance this with the necessity of the referral and may feel uncomfortable in their gatekeeper role, highlighting the complexity of shared decision-making, particularly when it comes to referrals [6, 37, 39].

GPs reported experiencing professional uncertainty leading them to refer to the ED, echoing previous research [16, 17]. Erring on the side of caution may be pronounced when dealing with small children whose condition can deteriorate quickly. Research has shown GPs may have less confidence with paediatric patients due to a lack of paediatric training or experience in treating children [2, 7, 14].

Previous studies have highlighted ED attendance is more frequent among lower socio-economic groups and those with lower levels of educational attainment [11]. This review supports this finding, demonstrating that across contexts GPs reported being more likely to refer those of lower socio-economic status [25, 29]. One study showed how U. S public funded insurance recipients, who may not be in urgent need for medical attention, were referred to ensure timely access to specialist services [31]. This indicates ED utilization by those from lower socio-economic backgrounds is influenced by system factors and clinical decision-making, and not just patient health-seeking behaviour.

Health system factors have been attributed to non-urgent use of the ED by healthcare professionals [37]. This review shows the lack of diagnostic equipment and treatments available in primary care may contribute to GPs referrals to the ED [26, 27]. Additionally, one study highlighted that lack of capacity in primary care resulted in approval of non-urgent visits to the ED [30]. Non-urgent use of ED during normal business hours has been documented previously, demonstrating the need for enhanced access to primary care in order to redirect non-critical care from emergency to primary care [38]. This is especially relevant to paediatric patients as children are high users of EDs, many with conditions that could be treated in primary care [3]. As health systems differ vastly across different countries and contexts with divergence of access across a range of public and private health systems, these findings suggest the need for an international study of GPs across health systems to understand the influence of the structure and financing of health systems on GP decision-making processes, and therefore on the pattern of paediatric presentations to the ED [7].

### Implications for policy and practice

Internationally, health systems are struggling to meet the demand on emergency departments, with healthcare planners and managers endeavouring to reduce the strain by eliminating non-urgent utilization [3, 4]. Strengthening primary care capacity and capabilities, through strategies such as increased supply and extended opening hours, can contribute to treating non-urgent cases in the community [3, 40]. This could address the pattern of referrals at certain times of the day and week, highlighted in this review. Enhanced paediatric training for GPs who experience professional uncertainty when treating children may lower referrals to the ED due to an aversion of risk. An alternative strategy could be the provision of remote consultations for GPs to seek advice from paediatricians. Stronger recognition of non-clinical factors and their impact on clinical decision-making is also essential during GP training. Greater awareness of various influences on clinical decision-making is vital to ensuring appropriate and excellent care for patients [41].

### Limitations

The findings of this review are limited primarily by the small number of included studies and the variation in study sample size. Literature on GP referral patterns is mostly based on referral to specialist services, and not to the emergency department, and often does not focus specifically on paediatric patients. The focus of this review was empirical studies and therefore policy documents have not been included, though it is accepted that this may shed further light on referral pathways.

## Conclusion

The decision to refer a child to the ED is imbued with a complex interplay of parent, GP and structural factors integral to the context upon which that decision is made. Enhanced awareness of non-clinical factors on referral decision-making is crucial to understanding patterns of paediatric unscheduled healthcare and to planning services that respond to parent’s and children’s needs, whilst allowing GPs to make decisions in the best interest of the child. Literature examining referral variation is rather dated, suggesting up to date research is required to account for system changes in recent years. We have identified scope for further research, such as qualitative research with GPs, which can contribute to understanding the inter-play between primary and emergency services, pertinent in the context of rising paediatric presentations to the ED.

## Supplementary information

**Additional file 1.** Key Terms & Boolean Operators.

**Additional file 2.** Quality Assessment Using Mmat^1^.

## Data Availability

Raw data was not generated for this systematic review. Data extracted and analysed in this study can be found in Table 2.

## Abbreviations

GP: General Practitioner
ED: Emergency department
MMAT: Mixed Methods Appraisal Tool
PRISMA: Preferred Reporting Items for Systematic Reviews and Meta-Analyses
U.S: United States
U.K: United Kingdom

## References

[1] Kleij K-S, Tangermann U, Amelung VE, Krauth C (2017). Patients’ preferences for primary health care–a systematic literature review of discrete choice experiments. BMC Health Serv Res.

[2] Kunin M, Turbitt E, Gafforini SA, Sanci LA, Spike NA, Freed GL (2018). General practitioner referrals to paediatric specialist outpatient clinics: referral goals and parental influence. J Prim Health Care.

[3] Cecil E, Bottle A, Cowling TE, Majeed A, Wolfe I, Saxena S (2016). Primary care access, emergency department visits, and unplanned short hospitalizations in the UK. Pediatrics..

[4] Baker R, Bankart M, Rashid A, Banerjee J, Conroy S, Habiba M (2011). Characteristics of general practices associated with emergency-department attendance rates: a cross-sectional study. BMJ Qual Saf.

[5] Bentley JA, Thakore S, Morrison W, Wang W (2017). Emergency department redirection to primary care: a prospective evaluation of practice. Scott Med J.

[6] Foot C, Naylor C, Imison C (2010). The quality of GP diagnosis and referral.

[7] Catzikiris N, Tapley A, Morgan S, Van Driel M, Spike N, Holliday EG (2019). Emergency department referral patterns of Australian general practitioner registrars: a cross-sectional analysis of prevalence, nature and associations. Aust Health Rev.

[8] Walsh B, Nolan A, Brick A, Keegan C (2019). Did the expansion of free GP care impact demand for emergency department attendances? A difference-in-differences analysis. Soc Sci Med.

[9] Turbitt E, Freed GL (2016). Paediatric emergency department referrals from primary care. Aust Health Rev.

[10] O’Donnell CA (2000). Variation in GP referral rates: what can we learn from the literature?. Fam Pract.

[11] Huntley A, Lasserson D, Wye L, Morris R, Checkland K, England H (2014). Which features of primary care affect unscheduled secondary care use? A systematic review. BMJ Open.

[12] Forrest CB, Nutting PA, Von Schrader S, Rohde C, Starfield B (2006). Primary care physician specialty referral decision making: patient, physician, and health care system determinants. Med Decis Mak.

[13] Newton J, Hayes VIC, Hutchinson A (1991). Factors influencing general practitioners’ referral decisions. Fam Pract.

[14] Allen AR, Turbitt E, Freed GL (2016). Use of home visiting GP services by paediatric patients presenting at emergency departments. Aust Fam Physician.

[15] Evans A (1993). A study of the referral decision in general practice. Fam Pract.

[16] Ingram JC, Calnan MW, Greenwood RJ, Kemple T, Payne S, Rossdale M (2009). Risk taking in general practice: GP out-of-hours referrals to hospital. Br J Gen Pr.

[17] Ringberg U, Fleten N, Førde OH (2014). Examining the variation in GPs’ referral practice: a cross-sectional study of GPs’ reasons for referral. Br J Gen Pr..

[18] Armstrong D, Fry J, Armstrong P (1991). Doctors’ perceptions of pressure from patients for referral. BMJ.

[19] Langley GR, MacLellan A, Sutherland HJ, Till JE (1992). Effect of nonmedical factors on family physicians’ decisions about referral for consultation. CMAJ Can Med Assoc J.

[20] van Suijlekom-Smit L, Bruijnzeels M, Van Der Wouden J, van der Velden J, Visser H, Dokter H (1997). Children referred for specialist care: a nationwide study in Dutch general practice. Br J Gen Pr..

[21] Moher D, Liberati A, Tetzlaff J, Altman DG (2009). Preferred reporting items for systematic reviews and meta-analyses: the PRISMA statement. BMJ..

[22] Rodríguez-Martín B, Nicholson E, McDonnell T, McAuliffe E. Factors influencing general practitioners decision-making to refer paediatric patients to the emergency department: a systematic review protocol. PROSPERO 2020 CRD42020145233 Available from: https://www.crd.york.ac.uk/prospero/display_record.php? ID=CRD42020145233. PROSPERO 2020 CRD42020145233 Available https://www.crdyork.ac.uk/prospero/display_record.php.IDCRD42020145233. 10.1186/s12875-020-01277-9 PMC7568398 33066729

[23] Covidence systematic review software VHI, Melbourne, Australia. Available at www.covidence.org.

[24] Pluye P, Robert E, Cargo M, Bartlett G, O’cathain A, Griffiths F, et al. Mixed methods appraisal tool (MMAT) version 2011. Propos Mix Methods Apprais Tool Syst Mix Stud Rev 2011;.

[25] Haimi M, Brammli-Greenberg S, Waisman Y, Baron-Epel O (2018). Physicians’ experiences, attitudes and challenges in a pediatric telemedicine service. Pediatr Res.

[26] Brousseau DC, Nimmer MR, Yunk NL, Nattinger AB, Greer A (2011). Nonurgent emergency-department care: analysis of parent and primary physician perspectives. Pediatrics..

[27] Orimadegun AE, Akinbami FO, Akinsola AK, Okereke JO (2008). Contents of referral letters to the children emergency unit of a teaching hospital, southwest of Nigeria. Pediatr Emerg Care.

[28] Dale J, Green J, Reid F, Glucksman E, Higgs R (1995). Primary care in the accident and emergency department: II. Comparison of general practitioners and hospital doctors. Bmj..

[29] Barwise-Munro R, Morgan H, Turner S. Physician and Parental Decision—Making Prior to Acute Medical Paediatric Admission. In Multidisciplinary Digital Publishing Institute; 2018. p. 117. 10.3390/healthcare6030117 PMC6165442 30227652

[30] Kini NM, Strait RT (1998). Nonurgent use of the pediatric emergency department during the day. Pediatr Emerg Care.

[31] Rhodes KV, Bisgaier J, Lawson CC, Soglin D, Krug S, Van Haitsma M (2013). “Patients who can’t get an appointment go to the ER”: access to specialty care for publicly insured children. Ann Emerg Med.

[32] Freed GL, Turbitt E, Kunin M, Gafforini S, Sanci L, Spike N (2016). General practitioner perspectives on referrals to paediatric public specialty clinics. Aust Fam Physician.

[33] O’Cathain A, Connell J, Long J, Coster J (2020). ‘Clinically unnecessary’use of emergency and urgent care: a realist review of patients’ decision making. Health Expect.

[34] Berry A, Brousseau D, Brotanek JM, Tomany-Korman S, Flores G (2008). Why do parents bring children to the emergency department for nonurgent conditions? A qualitative study. Ambul Pediatr.

[35] O’Cathain A, Connell J, Long J, Coster J. ‘Clinically unnecessary’use of emergency and urgent care: A realist review of patients’ decision making. Health Expect. 2019. 10.1111/hex.12995 PMC6978874 31663219

[36] Costet Wong A, Claudet I, Sorum P, Mullet E. Why do parents bring their children to the emergency department? A Systematic Inventory of Motivates Int J Fam Med 2015;2015:978412. 10.1155/2015/978412 PMC4649091 26618002

[37] Say RE, Thomson R (2003). The importance of patient preferences in treatment decisions—challenges for doctors. BMJ.

[38] O’Malley AS (2013). After-hours access to primary care practices linked with lower emergency department use and less unmet medical need. Health Aff (Millwood).

[39] Carlsen B, Norheim OF (2005). ‘Saying no is no easy matter’ A qualitative study of competing concerns in rationing decisions in general practice. BMC Health Serv Res.

[40] Guttmann A, Shipman SA, Lam K, Goodman DC, Stukel TA (2010). Primary care physician supply and Children’s health care use, access, and outcomes: findings from Canada. Pediatrics..

[41] Hajjaj FM, Salek MS, Basra MK, Finlay AY (2010). Non-clinical influences on clinical decision-making: a major challenge to evidence-based practice. J R Soc Med.

