# Supplementary material for: Factors influencing general practitioners decisions to refer Paediatric patients to the emergency department: a systematic review and narrative synthesis
Source: BMC Fam Pract. 2020 Oct 16;21:210. doi: 10.1186/s12875-020-01277-9 (PMC7568398; doi:10.1186/s12875-020-01277-9)
Supplement: Supplementary file 1 — Additional file 1. Key Terms & Boolean Operators. [file 12875_2020_1277_MOESM1_ESM.docx]

|  | **ADDITIONAL FILE 1: KEY TERMS & BOOLEAN OPERATORS** |
| --- | --- |
| **Medline** | (((("child"[MeSH Terms] OR "child"[All Fields]) OR ("pediatrics"[MeSH Terms] OR "pediatrics"[All Fields] OR "paediatric"[All Fields]) OR ("pediatrics"[MeSH Terms] OR "pediatrics"[All Fields] OR "pediatric"[All Fields]) OR ("infant"[MeSH Terms] OR "infant"[All Fields]) OR ("infant, newborn"[MeSH Terms] OR ("infant"[All Fields] AND "newborn"[All Fields]) OR "newborn infant"[All Fields] OR "neonate"[All Fields]) OR ("infant, newborn"[MeSH Terms] OR ("infant"[All Fields] AND "newborn"[All Fields]) OR "newborn infant"[All Fields] OR "newborn"[All Fields]) OR ("adolescent"[MeSH Terms] OR "adolescent"[All Fields])) AND ("decision making"[All Fields] OR ("Reason"[Journal] OR "reason"[All Fields]) OR refer[All Fields])) AND ("General Practitioners"[All Fields] OR ("physicians"[MeSH Terms] OR "physicians"[All Fields]) OR "Primary Care Physician"[All Fields] OR "General Practice Physician"[All Fields] OR "family physician"[All Fields])) AND ("Emergency Medical Services"[All Fields] OR "emergency care"[All Fields] OR "emergency department"[All Fields] OR "Emergency Health Services"[All Fields] OR "Urgent Care Center"[All Fields] OR "Urgent Care Centre"[All Fields] OR "accident and emergency"[All Fields]) |
| **CINAHL Plus,**  **EMBASE,**  **PsycINFO,**  **Web of Science** | (Child OR paediatric OR pediatric OR infant OR adolescent) AND (Decision making OR reason OR refer) AND (General Practitioners OR Physicians OR Primary Care Physician OR General Practice Physician OR family physician) AND (Emergency Medical Services OR emergency care OR emergency department OR Emergency Health Services OR Urgent Care Center OR accident and emergency) |
