# Supplementary material for: Factors influencing general practitioners decisions to refer Paediatric patients to the emergency department: a systematic review and narrative synthesis
Source: BMC Fam Pract. 2020 Oct 16;21:210. doi: 10.1186/s12875-020-01277-9 (PMC7568398; doi:10.1186/s12875-020-01277-9)
Supplement: Supplementary file 2 — Additional file 2. Quality Assessment Using Mmat1. [file 12875_2020_1277_MOESM2_ESM.docx]

| **ADDITIONAL FILE 2: QUALITY ASSESSMENT USING MMAT**^[[1]](#footnote-1)^  The 2011 version of MMAT was utilized which sets out 2 initial screening items and a further 19 items. There are four criteria for qualitative studies, which address source of data (1.1), analytical process (1.2), consideration to contextual setting (1.3) and researcher bias (1.4). Quantitative studies are divided into subgroups: randomized controlled trials, nonrandomized, and descriptive. For nonrandomized studies, the four criteria questions appraise selection bias (3.1), appropriateness of measurements (3.2), comparability (3.3) and completeness of outcome data and response rate (3.4). Descriptive quantitative studies are reviewed for sampling strategy (4.1) and representativeness of sample (4.2), appropriateness of measurements (4.3) and response rate (4.4). Scores of between 25% to 100% are awarded. | | | | | | | | | | | | | | |  |
| --- | --- | --- | --- | --- | --- | --- | --- | --- | --- | --- | --- | --- | --- | --- | --- |
| Study | Screening Questions | | Qualitative | | | | Quantitative | | | | | | | |  |
|  |  |  |  |  |  |  | Non-Randomized CT | | | | Descriptive | | | |  |
|  | S1 | S2 | 1.1 | 1.2 | 1.3 | 1.4 | 3.1 | 3.2 | 3.3 | 3.4 | 4.1 | 4.2 | 4.3 | 4.4 | Final score |
| Barwise-Munro et al., 2018** | Yes | Yes | Yes | Yes | Can’t tell | Can’t tell |  |  |  |  |  |  |  |  | 50 |
| Brousseau et al.,2011** | Yes | Yes | Yes | Yes | Can’t tell | Can’t tell |  |  |  |  |  |  |  |  | 50 |
| Dale et al., 1995** | Yes | Yes |  |  |  |  | Can’t tell | Yes | Yes | Can’t tell |  |  |  |  | 50 |
| Haimi et al., 2018** | Yes | Yes | Yes | Yes | Can’t tell | Can’t tell |  |  |  |  |  |  |  |  | 50 |
| Kini & Strait, 1998** | Yes | Yes |  |  |  |  |  |  |  |  | Can’t tell | Yes | Yes | No | 50 |
| Orimadegun et al., 2008*** | Yes | Yes |  |  |  |  |  |  |  |  | Yes | Yes | Can’t tell | Yes | 75 |
| Rhodes et al., 2013*** | Yes | Yes | Yes | Yes | Yes | Can’t tell |  |  |  |  |  |  |  |  | 75 |

1. Pluye P, Robert E, Cargo M, et al. Mixed methods appraisal tool (MMAT) version 2011. *Propos Mix Methods Apprais Tool Syst Mix Stud Rev*. Published online 2011. [↑](#footnote-ref-1)
